# PS-19 - ASSESSING THE IMPACT OF THE 2025/26 INFLUENZA VACCINATION PROGRAMME ON INFECTIONS AND HOSPITAL ADMISSIONS IN WALES

**DOI:** 10.1093/pubmed/fdag046.066

**Published:** 2026-07-09

**Authors:** Dr Warren Tennant, Caroline Harris, Panoraia Kalapotharakou, Jana Zitha, Richard Lewis, Jiao Song, Simon Cottrell

**Affiliations:** Public Health Wales; Public Health Wales; Public Health Wales; Public Health Wales; Public Health Wales; Public Health Wales; Public Health Wales

## Abstract

**Background:**

Seasonal influenza continues to place a substantial burden on healthcare systems in Wales, and vaccination remains the primary preventative strategy. Quantifying the population-level impact of vaccination, especially in reducing hospital admissions, helps inform planning and demonstrates the value of the annual vaccination programme.

**Objectives:**

To estimate the impact of seasonal influenza vaccination in Wales by quantifying reductions in infections and hospital admissions during the 2025/26 influenza season.

**Methods:**

We developed a mathematical model describing influenza infection, vaccination, and progression to hospital admission. The model was fitted to routinely collected surveillance data from primary care, including GP consultations and virological surveillance, alongside secondary care data on hospital admissions with recent laboratory confirmed influenza. Counterfactual scenarios—removing vaccination while holding other factors constant—were then simulated to estimate the number of infections and hospital admissions averted due to vaccination.

**Results:**

The model captured the observed trends in primary and secondary care for week 38 of 2025 through week 10 of 2026. Preliminary results estimated that vaccination reduced influenza infections by 18% (95% PI: [12–21%]) and averted 23% (95% PI: [18–27%]) hospital admissions over this period. The estimated impact varied across age groups, with the largest reductions observed among over 65-year-olds.

**Conclusions:**

These preliminary findings suggest that the 2025/26 influenza vaccination programme contributed to a meaningful reduction in infections and hospital admissions in Wales. The estimates will be refined as further data become available, and the results already highlight the benefits of sustained vaccination uptake and the value of impact modelling in supporting public health planning.

